# Impact of instrumental settings in electrospray ionization ion trap mass spectrometry on the analysis of multi-CH_3_-/CD_3_-isotopologs in cellulose ether analysis: a quantitative evaluation

**DOI:** 10.1007/s00216-021-03767-w

**Published:** 2021-12-16

**Authors:** Sarah Schleicher, Inka-Rosalia Lottje, Petra Mischnick

**Affiliations:** Institute of Food Chemistry, Technische Universität Braunschweig, Schleinitzstr. 20, 38106 Braunschweig, Germany

**Keywords:** Oligosaccharide ethers, Electrospray ionization ion trap mass spectrometry, Quantitative mass spectrometry, Substituent distribution, Methyl cellulose

## Abstract

Exact quantification of the molar ratios of isotopologous mixed *O*-methyl-*O*-methyl-*d*_*3*_-cellooligosaccharides (COS) comprising all combinations from fully methylated to fully deuteromethylated constituents within an individual degree of polymerization (DP) is the key step in the analysis of the substituent distribution over the polymer chains in methyl celluloses (MC). Deuteromethylation of MC is performed to level chemical differences, but due to a *m/z* range of 3 DP·ΔMe/Me-*d*_*3*_, bias during MS measurement cannot certainly be excluded. Therefore, ionization, ion transportation, and ion storage were studied with an electrospray ionization ion trap mass spectrometer (ESI-IT-MS) using binary equimolar mixtures of per-*O*-Me- and per-*O*-Me-*d*_*3*_-COS, defining the border cases of a particular Me/Me-*d*_*3*_-profile. Reference data of their molar ratio were determined after reductive amination with *m*-amino benzoic acid by HPLC-UV. COS of DP2–6 were measured as their sodium adducts at *c* = 10^−6^ M by syringe pump infusion. The impact of the RF voltage of the ion trap (TD), the octopole RF and DC voltages, and the *Cap Exit* potential on absolute and relative ion intensities were studied. Adapting the *Cap Exit* voltage was essential for correct quantification of DP2, while all COS of higher DP behaved insensitive with respect to bias. To check whether any bias occurs in the electrospray ionization process of the isotopologs, concentration-dependent measurements were performed with optimized instrumental settings for each DP. Intensity ratios IR = *I* (Me-*d*_*3*_)/*I* (Me) did not show any concentration-dependent trend and no selective ion suppression. Its decrease with DP observed under usually applied standard conditions (*smart mode*) is a consequence of discrimination according to *m*/*z* and can be overcome by appropriate instrumental settings of Oct 2 DC and TD. IR between 0.971 ± 0.008 and 1.040 ± 0.009 with no trend for DP (2-6) were obtained by averaging all measurements in the range 2 · 10^−7^ to 2 · 10^−5^ M total concentration. The DP-related optimized settings were applied to two MCs and compared with the results obtained under so far applied standard conditions.

**Graphical abstract:**

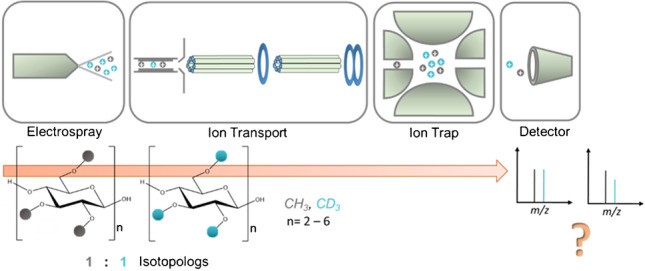

**Supplementary Information:**

The online version contains supplementary material available at 10.1007/s00216-021-03767-w.

## Introduction

The quantitative determination of the concentration of analytes is mainly performed by isotope dilution mass spectrometry (IDMS). This method is based on the assumption that the physical and chemical behaviors of two isotopologs are identical as long as the linkages of the considered isotopes are not involved in transformations (kinetic isotope effect). Consequently, they will undergo the same discriminations during sample treatment and workup, and will show the same sensitivity in ionization and MS analysis. Thus, adding an exactly known amount of a defined (certified) isotopolog reference material to a known amount of the sample material containing the analyte finally allows for quantification of the latter by comparison of their MS signals. In the case of macromolecules, e.g., proteins or polysaccharides, this approach cannot be applied directly [1]. Nevertheless, beside matrix-assisted laser desorption ionization time-of-flight mass spectrometry (MALDI-ToF-MS), electrospray ionization coupled with mass spectrometry (ESI-MS) is the key analytical technique in the field of quantitative pattern analysis of polysaccharide ethers [2, 3].

**Fig. 1:**
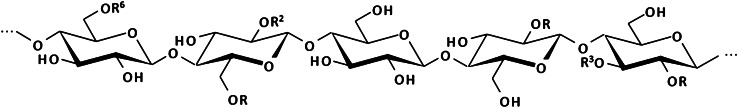
General structure of cellulose ethers (R = –CH_3_, –CH_2_CHROH/CH_3_, –CH_2_COONa)

Cellulose ethers are, on a quantity basis, the most important cellulose derivatives. Concerning their rheological, emulsifying, gelling, thickening, and film-forming properties, they find a wide field of application in food, cosmetics, and pharmacy, as well as textile and construction [4]. Cellulose ethers are obtained by partial functionalization of cellulose with mainly methyl, hydroxyalkyl, or carboxymethyl groups (Fig. 1). The extent of transformation is expressed by the average degree of substitution (DS), i.e., the number of modified OH groups per glucosyl unit. Beside the number, the pattern of substituents in a glucosyl unit varies with respect to the positions etherified, O-2, O-3, and O-6 [2].

Amongst others, the physicochemical properties notably depend on the DS as well as on the distribution of the substituents along and over the cellulose chains. To characterize the substituent distribution in such complex semi-synthetic polymers, cellooligosaccharides (COS) obtained by partial depolymerization are separated according to their degree of polymerization (DP), and within each DP according to their number of substituents and consequently various masses, or more exactly *m/z*, by MS. Since exact quantification of the molar ratios of the constituents contributing to the substituent pattern profiles in such diades, triades, tetrades, etc. is essential for any conclusions on their homo- or heterogeneity, bimodal or block-like structures [1, 5], reliability of the MS results is of great importance.

It should be emphasized that this type of analysis does not aim for absolute concentration data, but the determination of the relative molar ratios of all constituents belonging to the COS of a particular DP [1]. With each additional substituent introduced, the chemistry, especially polarity and thus surface activity, and mass of an oligosaccharide change. In order to prevent discrimination effects, peralkylation of the entire material is carried out prior to the partial depolymerizaton to COS. In the case of MC, this is performed with iodomethane-*d*_*3*_ in order to finally generate chemically more uniform, fully 2,3,6-*O*-alkylated oligosaccharides [2, 6]. Thus, all combinations and regioisomers of (*O*-Me,*O*-Me-*d*_*3*_)_3DP_-COS are obtained which (within each DP) are isotopologs. This approach using deuterated alkyl groups for peralkylation of a polysaccharide ether can be considered as “internal isotope labeling.” At the same time, Δ*m/z* is reduced from 14 to 3 for the functional groups in the glucosyl units, and the maximal Δ*m/z* range for a set of COS derivatives belonging to a particular DP decreases from 42·DP to 9·DP.

However, ^1^H and ^2^H (D) are an exceptional pair of isotopes, since the relative increase in mass is 100%, and size and physical properties are by far more different than for ^12^C/^13^C or ^14^N/^15^N. And in contrast to carbon, H is usually found in the periphery of molecules, explicitly in our case of *O*-methylated carbohydrates. Therefore, a greater impact on surface properties can be expected. The vapor pressures of deuterated alkanes show an inverse isotope effect, i.e., a larger vapor pressure of the heavier deuterated isotopolog [7]. The physicochemical differences of C-H and C-D are also clearly visible from the retention behavior in chromatography: In gas chromatography on common non-polar polysiloxane phases, deuteromethylated compounds are less retained than their methylated analogs [8, 9], and the same is observed in reversed-phase liquid chromatography [5], while the behavior on normal-phase LC columns is the opposite. Thus, a highly deuteromethylated molecule shows less interaction with a non-polar phase than its methylated counterpart. This seems to point to higher polarity compared to the CH_3_ isotopologs, or perhaps to a smaller size and hence less surface available for interaction with the stationary phase. And indeed, due to its larger mass, the zero-point vibration of D is located at lower energy compared to H. In combination with the typical anharmonic nature of the C-H vibration, this results in a shorter equilibrium distance of C-D compared to C-H [7]. The C-D linkage is stronger by about 5%. Consequently, the van der Waals radius of CD_3_ is shorter than that of CH_3_. This volume effect is small, but accumulates in heavily *O*-methylated compounds.

Electrospray ionization (ESI) of alkylated carbohydrates usually generates sodium adducts, since the many oxygen atoms can nicely coordinate to the alkali ion. Alternatively, charged tags can be introduced [6, 10]. In this paper, sodium adducts shall be considered. For quantitative evaluation of the isotopologs’ signals, the equilibrium constants for [M+Na]^+^ formation should be equal [11, 12]. The coordination ability is influenced by the number, orientation, and basicity of the oxygens, which might be slightly different. Furthermore, ion sensitivity in the ESI process depends on surface activity, and in this context also on molecule volume and surface size [13]. A higher surface activity of one analyte enhances the chance to compete with other analytes for covering the droplet surface, thus showing the larger equilibrium constant for this partition [13–16]. Competition of analytes for the bulk and the droplet surface becomes especially relevant at and beyond surface saturation of the droplets.

**Fig. 2:**
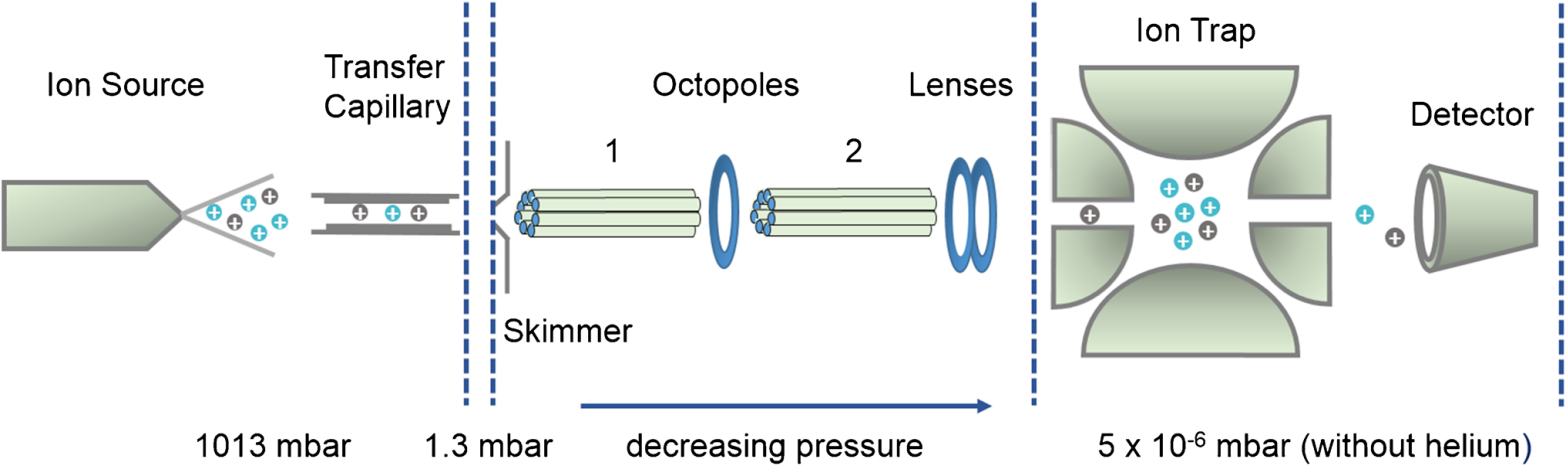
Instrumental set-up of the ESI-IT-MS used in this study (according to the information for the HCT Ultra ETD II, Bruker Daltonics GmbH & Co. KG, Bremen, Germany)

Methyl and methyl-*d*_*3*_ necessarily differ in mass. Therefore, for a relative quantification, it has to be ensured that there are no discrimination effects during the analysis—including the ionization, ion transportation, and mass analyzer unit as well as the detector. In addition to potential sources of bias during the ESI process, mainly affected by the chemistry of the analytes and matrix effects as just discussed, there are some instrumental settings, which can cause discrimination of ions according to their *m*/*z* during ion transportation from the ion source to the mass analyzer, and with regard to storage in the ion trap and finally detection. Kruve et al. reported on such optimization for pesticide analysis [17]. To demonstrate this, the ESI-IT-MS used in this study (HCT Ultra ETD II, Bruker), is schematically shown in Fig. 2.

An ESI-IT-MS device can be divided into four areas: the ionization unit; the transportation unit, consisting of the transfer capillary and the skimmer as well as the octopoles and lenses; and finally, the ion trap followed by the detector. In the case of the Bruker HCT Ultra ETD II, the nebulizer needle is grounded. The instrument can be operated in two different modes. The so-called *smart mode* allows convenient operation of the device by the user. Voltages of the transportation and focusing unit and of the mass analyzer are indirectly controlled. One smart parameter is the so-called *Target Mass* (TM) which optimizes the intensity of the ion current in the range of the selected mass (i.e., *m*/*z*). It has an impact on the octopole voltages Oct 2 DC, Oct RF, and the *Trap Drive* (TD) voltage. This is the amplitude of the RF voltage applied to the ring electrode and affects the storage of ions in the trap. A further parameter called *Compound Stability* (CS) adjusts the voltage at the exit at the transfer capillary (*Cap Exit*) in dependence on the chosen TM. Larger voltages promote the dissociation of clusters or of multiply charged ion adducts, but can also cause fragmentation of labile ions [18–20]. Thus, this setting is of interest in our study of sodium adducts. Finally, the *Trap Drive Level* (TDL) can be set in the *smart mode*, as for CS, with values in the range of 10–1000%. By the TDL, the slope of the TD increases depending on the TM and is adjusted, i.e., the higher the TM, the larger the slope. The supporting information gives an overview on how the smart parameters control these various interrelated voltages (s. Supplementary information (ESM) Fig. S1–S3).

On the contrary, in the so-called *expert mode*, the settings of the transportation unit and the ion trap can be adjusted directly and largely independently. These are the voltages of the skimmer, the *Cap Exit*, the DC and RF voltages of the octopoles (Oct 1 and Oct 2 DC; Oct RF), the voltage of the lenses, and the RF voltage applied to the ring electrode of the ion trap (TD). The initial setting of Oct 1 DC is 8 V and should only be changed if necessary. In contrast, the voltage of the end plate is fixed at − 500 V (positive mode) and cannot be varied. The role of the individual voltages for a similar quadrupole ion trap ESI-MS (Agilent) has been reported by Kruve et al. [17]. They will be discussed below when presenting the results.

The highest impact on the ion intensity is related to TD (dimensionless quantity related to the amplitude of the RF voltage of the ion trap), the DC voltage at the second octopole (Oct 2 DC) as well as the RF voltages of both octopoles (Oct RF), and finally the voltage which is applied to the end of the transfer capillary (*Cap Exit*). Therefore, in our studies dealing with potential discrimination effects during a relative quantification of isotopolog analytes, we focused on these settings.

In order to study the accuracy and precision of the entire process, from ion formation over the ion transportation to the storage in the trap, we prepared equimolar binary mixtures of the two confining analytes of a particular DP, the fully 2,3,6-*O*-methylated and fully 2,3,6-*O*-deuteromethylated COS from DP2–6 (s. ESM Fig. S4). These isotopologs are the boundary structures of all possible substitution patterns of each DP. If no discrimination effects with respect to the relative quantification occur for these analytes presenting the largest possible difference in mass and chemistry of a particular DP, it should be possible to quantify the molar ratios of all isotopologs in between and thus determine the distribution of substituents over the entire mass range. Their absolute concentration and exact molar ratio (MR) were determined by HPLC/UV after labeling with *m*-amino-benzoic acid (*m*ABA). First, the influence of the RF voltage in the IT (TD) on the molar ratio of detected ions (IR (Me-*d*_*3*_/Me)/MR (Me-*d*_*3*_/Me) was investigated systematically. Afterwards, the voltages affecting the ion transportation were varied. Since larger COS are prone to double-charged ion formation, the impact of *Cap Exit* on ion ratios was also studied. Since bias in the ESI process should become visible above the saturation point (expected at about 10^−5^ M), total concentrations of the binary mixtures were varied up to six orders of magnitude. Finally, it was checked whether the observed effects play a role in the determination of the methyl distribution of real MC samples.

## Materials and methods

### Materials

The starting material cellulose acetate with 40 % acetyl groups was purchased from Fluka. All chemicals were purchased from Sigma-Aldrich/Merck with the following purities: iodomethane (≥ 99%), deuterated iodomethane (≥ 99.5 atom% D), 2-picoline borane complex (≥ 95%), *m*-aminobenzoic acid (*m*ABA) (≥ 99%), and acetic acid, HOAc (≥ 99.8%), methyllithium solution (1.6 M in diethyl ether). Trifluoroacetic acid (≥ 99.9%) as well as DMSO (≥ 99.5 % for synthesis) was from Roth.

Both MC samples—MC1 (DS 1.29) and MC2 (DS 1.96)—had been provided by former DOW Wolff Cellulosics GmbH, Bomlitz, Germany. Solvents were from Riedel-de Haen (acetonitrile (ACN), methanol (MeOH)) and Fisher Chemicals (dichloromethane (DCM), toluene). For ESI-MS measurements, LC-MS grade was used, and for all other applications, HPLC grade.

## *O*-Methyl- and *O*-methyl-*d*_*3*_-cellooligosaccharides (Me-COS, Me-*d*_*3*_-COS)

*O*-Methyl- and *O*-methyl-*d*_*3*_-cellooligosaccharides of DP2–6 were obtained by semi-preparative HPLC separation of hydrolyzed per-*O*-methylated and per-*O*-deuteromethylated cellulose, respectively. The latter were synthesized from 1.0 g cellulose acetate in DMSO according to Ciucanu and Kerek [21] with NaOH/MeI and MeI-*d*_*3*_, respectively. Afterwards, the samples were dialyzed against water and freeze-dried. Completeness of alkylation was checked by the absence of OH absorption > 3000 cm^−1^ by ATR-IR spectroscopy. Unless complete alkylation was achieved, alkylation was repeated by a modified Hakomori method using Li-dimsyl in DMSO as base [5, 22]. Li-dimsyl was prepared by addition of the required volume of methyllithium solution to the same volume of dry DMSO under nitrogen.

### Partial hydrolysis

Overall, 400 mg of per-*O*-deuteromethylated cellulose ether and 300 mg of methylated cellulose ether were partially hydrolyzed. Therefore, permethyl celluloses (8 mg/portion) were swollen overnight in a 5-mL V-Vial in 4.25 mL of a mixture of acetone/water (50/50, v/v). For the partial hydrolysis, 0.75 mL TFA (final concentration in the vial was 2 M) was added and the sample was heated at 120 °C for 22 min. After cooling to room temperature, aqueous acid was removed in a stream of nitrogen, co-distilled with toluene, and finally evaporated to dryness. The obtained 2,3,6-*O*-methylated/ deuteromethylated COS were unified and dissolved for the fractionation in H_2_O/ACN (80/20, v/v) to a concentration of 60 mg mL^−1^.

### Fractionation

Hydrolysates containing individual Me- and Me-*d*_*3*_-COS were separated by semi-preparative HPLC equipped with a fraction collector (all components are from the Agilent series Infinity 1260). The separation was carried out on a RP-C_18_-column (Phenomenex, Gemini 5 μm, 250 × 10 mm) at a flow rate of 2 mL min^−1^ with H_2_O + 1% HOAc (A) and ACN + 1% HOAc (B) as mobile phase, starting with 80% A and reaching 0% A at 50 min. The injection volume was 100 μL (6 mg COS per run). Retention times of the non-UV-active COS ethers and the maximum separation capacity of the column were determined using an ELSD detector (Softa 1400 ELSD). The fractionation itself was carried out without any detector. From the collected fractions (DP2–6), ACN was evaporated, and the remaining aqueous phases were saturated with NaCl and extracted several times with DCM. The combined DCM phases were dried with Na_2_SO_4_ and afterwards evaporated to dryness.

### Binary mixtures of Me and Me-*d*_*3*_-COS

From the COS fractions, standard solutions were prepared for each DP at a concentration of 2.5 · 10^−3^ M (DP2 and 3) and 2.5 · 10^−4^ M (DP4–6), respectively. The exact concentration of the COS ether of interest was determined by HPLC-UV after reductive amination with *m*ABA in triplicate. Each sample was injected three times.

Calculated volumes of unlabeled standard solutions of Me- and Me-*d*_*3*_-COS of the same DP were mixed in order to prepare equimolar binary standard mixtures with a total concentration of 2 · 10^−3^ M (DP2 and 3) and 2 · 10^−4^ M (DP4–6), respectively. The exact molar ratio of these mixtures was also determined in triplicates by HPLC-UV after labeling with *m*ABA. Each sample was injected three times. The molar ratio was calculated as weighted average as follows:

1 $$\overline{x}=\frac{\sum_{i=1}^n{p}_i{x}_i}{\sum_{i=1}^n{p}_i}$$

*x*_*i*_: average of the individual series of measurements

*n*: number of series of measurements; *n* = 3

*p*_*i*_: reciprocal square of the standard deviation of the average values *x*_*i*_; *p*_*i*_ = 1/*s*_*i*_^2^

An internal and external standard deviation was determined for the weighted average:

2 $${s}_{{int}}^2=\frac{1}{\sum_{i=1}^n{p}_i}$$

3 $${s}_{{ext}}^2=\frac{1}{n-1}\ \frac{\sum_{i=1}^n{p}_i\left(x_{i}-\overline{x}\right)^{2}}{\sum_{i=1}^n{p}_i}$$

According to the convention, the larger error is always given as the average standard deviation of a weighted average. In our case, the internal mean error according to (2) was always larger.

For MS studies, the binary mixtures were diluted with MeOH stepwise (factor 10/step) up to 2 · 10^−9^ M.

### Quantitative determination of the concentration and the molar ratio of Me-*d*_*3*_- and Me-COS by HPLC/UV after reductive amination

In order to determine the exact concentration of the standard solutions, 200 nmol of DP2 and 3 and 20 nmol of DP4, 5, and 6 were labeled. Therefore, a defined volume of these solutions was transferred to a 1-mL V-vial and MeOH was added up to a total volume of 0.5 mL. To the solution, 0.3 mL *m*ABA (containing 4 eq per reducing end in MeOH) and 0.15 mL HOAc were added. The mixture was heated to 40 °C for 30 min. Subsequently, 50 μL of 2-picoline borane was added (equimolar to *m*ABA) and the mixture was heated to 40 °C for 45 min. Subsequently, the solvent was removed in a stream of nitrogen at ambient temperature. The labeling procedure was repeated two more times. Finally, the residue was dissolved in 1 mL (DP2 and 3: 200 nmol mL^−1^) or 0.2 mL MeOH (DP4–6: 100 nmol mL^−1^), respectively. Completeness of labeling was checked by ESI-IT-MS in positive and negative modes.

The procedure was the same for the binary mixtures. For DP2 and 3, 400 nmol, and for DP4–6, 40 nmol were labeled. The residue was dissolved in 1 mL (DP2 and 3: 200 nmol mL^−1^ per isotopolog) or 0.2 mL MeOH (DP4–6: 100 nmol mL^−1^ per isotopolog), respectively.

#### HPLC-UV

HPLC/UV analysis was performed on an Agilent Infinity 1260 instrument. *m*ABA-labeled COS were measured at 254 nm after separation on an RP-C18-column (Phenomenex, Gemini, 250 × 4.60 mm, 5 μm) and a flow rate of 0.8 mL min^−1^. A gradient system was used, consisting of water (A) and ACN (B) with 1% HOAc as eluent, starting at 80% A with linear decrease to 0% A within 50 min. Quantitative analysis for DP2 was performed at 45 °C and for DP5 at 35 °C. COS of all other DPs were measured at 22 °C. The injection volume was 10 μL for DP2 and 3 as well as for the calibration samples and 25 μL for DP4–6. Quantification was performed with *m*ABA-labeled 2,3,6-tri-*O*-methyl-glucose as external standard.

In order to exclude any coelution of impurities in the binary mixtures, which might occur due to the mixing of the two standard solutions, one of the *m*ABA-labeled samples of the binary mixtures as well as the standard solutions were measured additionally by ESI-MS (Bruker HCT Ultra ETD II). If necessary, for instance for DP2, HPLC conditions were adapted to achieve full separation of the peaks of interest.

Separation was performed on a C18 column (Phenomenex Kinetex RP18, 100 × 2.1 mm, 2.6 μm) with a gradient system consisting of water (A) and ACN (B) with 1 % acetic acid, starting at 90% A with a linear decrease to 20 % A within 30 min and a flow rate of 0.2 mL min^−1^.

The MS measurement parameters were as follows: Nitrogen was used as drying gas (10 L min^−1^, 365 °C) and nebulizer gas (50 psi), capillary + 4.5 kV, end plate offset − 500 V, skimmer − 40 V, *Cap Exit* − 280 V, Oct 1 DC − 8 V, Oct 2 DC − 2.70 V, Oct RF 200 Vpp, lens 1 5 V, lens 2 60 V, *Trap Drive* 107.8, and ion charge control 70,000 as well as maximum accumulation time of 200 ms. Injection volume: 2–4 μL.

### MS measurements of binary mixtures

HCT Ultra ETD II (Bruker Daltonics, Bremen, Germany), equipped with an ion trap (IT) and a conversion detector with a photomultiplier (delay detector), was employed. Me-COS/Me-*d*_*3*_-COS sample solutions in MeOH were applied by syringe pump infusion at a flow rate of 200 μL h^−1^. Nitrogen was used as drying gas (6 L min^−1^, 300 °C) and nebulizer gas (10 psi). Data were recorded in positive ion mode. The following parameters were used: capillary − 3.5 kV, end plate offset − 500 V skimmer 40 V, Oct 1 DC 8 V, lens 1 − 5 V, lens 2 − 60 V, and ion charge control 100,000.

Binary mixtures (*c* ≈ 2 · 10^−6^ M) of DP2–6 were measured with independent variation of TD and Oct 2 DC, as well as *Cap Exit*, in a stepwise manner (s. ESM, Fig. S1–S3 and Tables S1 and S2, respectively). Exact measurement parameters of the individual experiments (TD, Oct 2 DC as well as *Cap Exit*) are mentioned in “Results and discussion.” For each measurement, 200 scans were accumulated. Measurements were recorded in triplicate.

To check discrimination effects during the ionization, a concentration series (2 · 10^−9^–2 · 10^−3^ M) of the binary mixtures of each DP was measured. Each dilution was measured five times, and 200 scans were accumulated for each measurement.

All mass spectral data were analyzed by Bruker Daltonics Data Analysis software. Peak intensities of the mass spectra were corrected for the corresponding isotopic signals by adding their calculated intensities to the main peak after a noise correction. The isotopolog distribution was calculated with the program *Isotope Distribution Calculator* (IDCalc, by Michael J. MacCoss, Department of Genome Sciences, University of Washington) which allows consideration for residual H in the applied MeI-*d*_*3*_. The experimentally observed [M-1]-isotope signals for each DP were used to approximate its purity. The best approximation was achieved for a purity of 99.87 atom% D, which was used for the calculation of the isotope distribution. Beside sodium adducts, the analytes were detected as potassium (≤ 8% relative abundance referred to the [M+Na]^+^ main peak) and lithium (≤ 8%) adducts as well as the sodium adducts clustered with TFA-salts of sodium (≤ 15%) and lithium (≤ 10%). The extent depends on the measurement parameters, especially on *Cap Exit*, and may vary for individual sample solutions. Mentioned amounts refer to the [M+Na]^+^ main peak at *Cap Exit* 280 V. For the evaluation, only the sodium adducts were considered. Both isotopolog analytes within binary mixtures showed very similar complexation behavior.

### Application of the optimized MS parameters to MCs

Deuteromethylation of the free OH groups of MC1 and 2 were performed as described above. For the partial hydrolysis, 10 mg of each material was weighted in a 5-mL V-Vial and swollen in 4.25 mL of a mixture of water and acetone (50/50, v/v) overnight. Then, 0.75 mL TFA (final concentration in the vial was 2 M) was added and the sample was heated at 120 °C for 22 min. After cooling to room temperature, aqueous acid was removed in a stream of nitrogen at ambient temperature, co-distilled with toluene, and finally evaporated to dryness. The residue was dissolved in 2.5 mL of MeOH (LC-MS).

To determine the substituent distribution, the samples were diluted to a concentration of 0.05 mg/mL and were infused to ESI-IT-MS by syringe pump at a flow rate of 200 μL h^−1^. For the general measurement parameters, see “Mass Spectrometry measurements of binary mixtures.” MS spectra were recorded with the optimized parameters (Table 2) and with the standard parameters (TM (*m*/*z*) 1000; TDL 100 %; CS 1000%—*Cap Exit* 280 V; Oct 2 DC 2.7 V; Oct RF 200 Vpp), usually applied by us for real MC samples. All methyl distribution profiles were evaluated as described previously [6].

## Results and discussion

### Preparation of equimolar Me-d_3_/Me-COS mixtures and reference analysis

First, 2,3,6-*O*-methylated cellooligosaccharides (Me-COS and Me-*d*_*3*_-COS) from DP2 to 6 were prepared from the corresponding permethylated MCs by partial hydrolysis and semi-preparative HPLC. Standard solutions of each COS were prepared at the concentration of about 2.5 · 10^−3^ M in MeOH (DP2 and 3) and 2.5 · 10^−4^ M (DP4–6), respectively. The absolute concentrations of the respective COS in these standard solutions were determined after labeling with *m*ABA by HPLC-UV. The peak purity was checked by LC-ESI-MS. Minor amounts of undermethylated COS of the same, but preferentially the next higher DP, as well as terminally 4-*O*-methylated COS of the same DP, but mainly the next-lower DP, were detected. This control was important to be aware of any coelution with the main constituents which would falsify the reference data obtained by HPLC-UV and thus impair the evaluation of the MS data. Based on these data, a tentatively equimolar mixture of each DP of COS was prepared, and their total concentration and exact molar ratio (MR: c (Me-*d*_*3*_-COS*)*/c (Me-COS), briefly Me-*d*_*3*_/Me) was determined. The MR and respective standard deviations (SD) are summarized in Table 1. The precision of MR values is very good. Since any error or loss should affect both analytes in the same way, we also conclude high quality of accuracy. The determination of the absolute concentrations was less precise. Fortunately, very high accuracy of absolute concentrations is not critical for the study.

**Table 1:** Absolute concentrations of binary standard solutions and MR of Me-*d*_*3*_-COS/Me-COS in individual binary mixtures, determined after reductive amination with *m*ABA (*n* = 3) by HPLC/UV (*n* = 3), and standard deviation (SD); individual and total concentrations × 10^−4^ M

**COS**	**DP2**	**DP3**	**DP4**	**DP5**	**DP6**
Me-*d*_*3*_	10.91	14.42	1.15	1.11	1.07
Me	10.08	14.36	1.18	1.11	1.10
Total	20.98 ± 4.55%	28.77 ± 4.43%	2.33 ± 8.16%	2.22 ± 5.29%	2.16 ± 2.28%
MR	1.082 ± 0.002	1.007 ± 0.000	0.971 ± 0.001	0.999 ± 0.003	0.971 ± 0.003

The exact MR were later used to normalize all ESI-MS data in order to gain the ion intensity ratio for an equimolar mixture (IR (Me-*d*_*3*_/Me)/MR (Me-*d*_*3*_/Me).

### Influence of the Trap Drive on the ion storage

As outlined in the “Introduction,” this study focusses on three critical aspects: the formation of ions in the ESI source, the transportation of ions via the transfer capillary and the octopoles, and the storage in the trap (s. Fig. 2). We did not consider the detector in our studies. The number of emitted secondary ions depends on the speed of the ions. In our case, the methylated isotopolog is around 2% faster compared to the deuteromethylated one. Therefore, any potential bias caused by this small difference can probably be neglected. According to Kruve et al., MS settings should be optimized with respect to optimal ion intensity and thus sensitivity along the pathway of the ions [17]. While a high sensitivity is generally expedient, in our case, not the absolute intensity but the prevention of any discrimination of one of two isotopologs against the other one is the primary goal.

When ions reach the trap, they possess a comparatively large kinetic energy which depends on the applied potential gradient and prevents them from being trapped. Therefore, they are usually damped through elastic collisions with a buffer gas (helium) [23]. The applied main RF voltage induces a secular motion. As a result, ions of a particular *m*/*z* move on stable trajectories in the trap. The larger the amplitude and the smaller the *m*/*z* of the ion, the stronger is the induced motion [10, 24]. Storage of ions in the trap is only possible if the trajectories are stable in both radial and axial directions (*r*- and *z*-) at the same time. To achieve stabile trajectories, the amplitude as well as the phase of the RF voltage must be appropriate, when the ions enter the trap [25]. The Mathieu parameters *a*_*z*_ and *q*_*z*_ can be used to determine the occurrence of stable trajectories in *r*- and *z*-directions depending on the *m*/*z* of the ion [26].

4 $${a}_z=-2{a}_r=\frac{-16 eU}{m{\Omega}^2\left({r}_0^2+2{z}_0^2\right)}$$

5 $${q}_z=-2{q}_r=\frac{8 eV}{m{\Omega}^2\ \left({r}_0^2+2{z}_0^2\right)}$$

In the case of an ion trap, only the *q*-parameter is used (normally, there is no DC voltage applied to the ring electrode; therefore, *a*_*z*_ = 0) [10]. As can be seen from Eq. (5), the parameter *q*_*z*_ depends on the amplitude *V* and frequency *Ω* of the RF voltage (which is fixed) as well as on the charge *e* (corresponding to *n · z*)*,* and mass *m* of the ion. Stable ion trajectories can be obtained in the range from *q*_*z*_ = 0 to 0.908. If *q*_*z*_ exceeds this limit (for example by ramping the RF voltage), the secular motion induced by the applied RF voltage increases such that trapping is no longer possible [10].

Due to the batch-wise operation mode of a 3D ion trap at a particular constant RF voltage and the highly pressure gradient, less than 5 % of the total ions are collected [25, 26]. Therefore, it is prone to mass discrimination effects.

In order to study how the TD (specifying the amplitude of RF voltage at the IT) affects the individual ion intensities and whether a significant bias is observed related to Δ*m/z* of our isotopologous analyte pairs, the binary mixtures of Me-*d*_*3*_-COS and Me-COS in MeOH at a total concentration of about 2 · 10^-6^ M (compare to Table 1) were measured at various TD voltages. For the measurements, we initially choose the instrumental settings commonly applied for the measurement of such COS derivatives in MC analysis (for ESI, see experimental: MS: *Cap Exit* 280 V, Skimmer 40 V, Oct 1 DC 8 V, Oct 2 DC 2.7 V, Oct RF 200 Vpp).

**Fig. 3:**
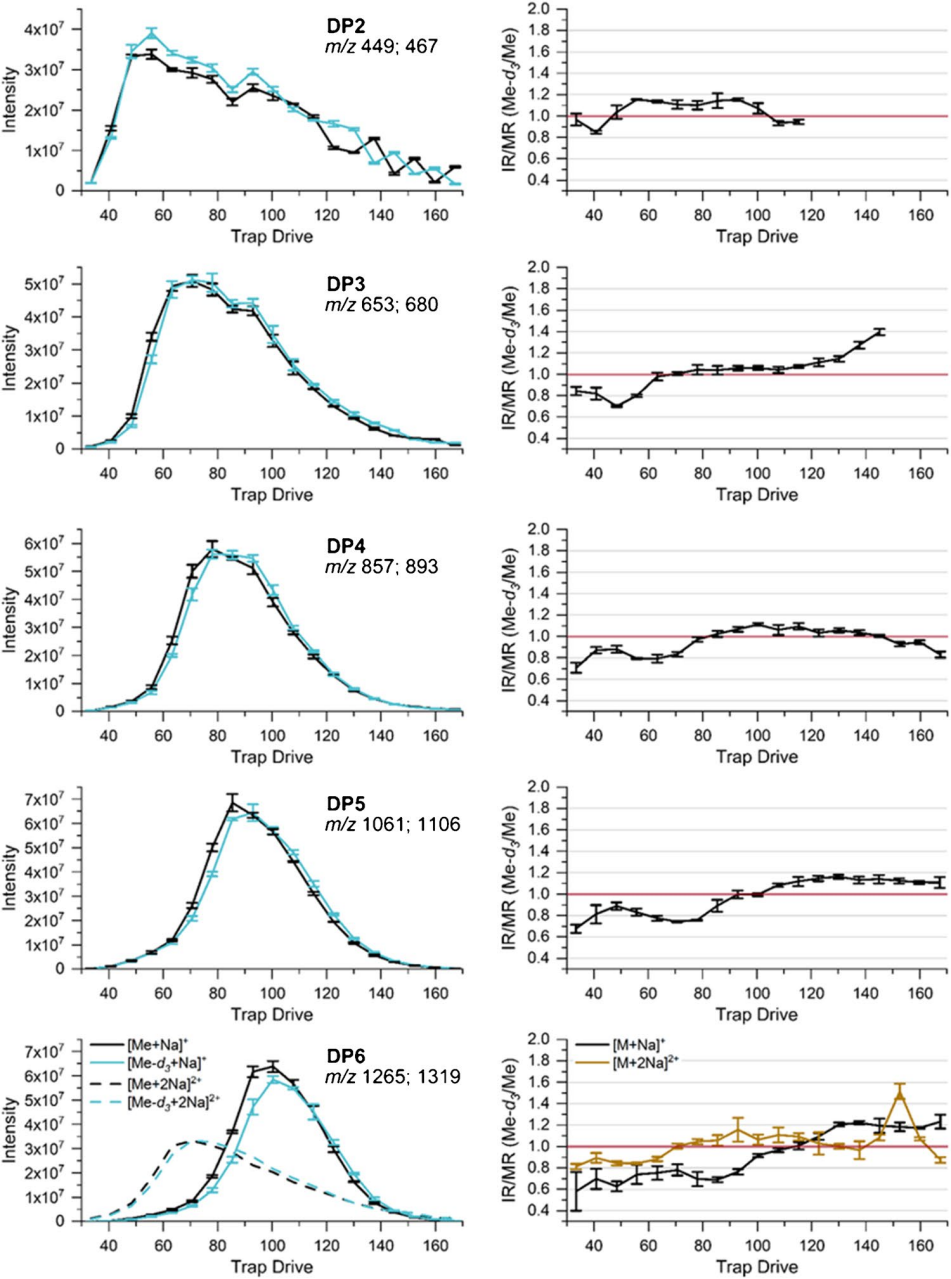
Left: absolute intensities recorded for the binary mixtures of COS (Me and Me-*d*_*3*_) at a total concentration of 2 · 10^−6^ M in MeOH by ESI-IT-MS (syringe infusion) at various *Trap Drive* (RF amplitude of the ring electrode). Right: calculated intensity ratio (IR). Data are corrected for the exact molar ratio (MR) according to the reference data given in Table 1 to represent an equimolar mixture. Further measurement parameters are given in Table 2, *n* = 3

The results are shown in Fig. 3. In the first column, the recorded intensities are shown for each COS of DP2–6. For DP5 and DP6, in addition, double-charged adducts were detected. However, for DP5, we did not consider them here because of the very low intensities and its discrimination due to much lower *m*/*z*. The intensities were adapted to an equimolar mixture. The second column shows the corresponding normalized ratio of ion intensities IR (Me-*d*_*3*_/Me)/MR (Me-*d*_*3*_/Me).

Generally, the ion intensities first increase with TD, go through a maximum, and then drop again. Beyond the RF voltage related to the maximum ion intensity, the secular motion becomes too strong for an effective storage. A comparison of the curves obtained for Me-*d*_*3*_-COS and Me-COS shows a slight distortion of the profile including a little shift of the intensity maxima to larger TD for the heavier Me-*d*_*3*_-compounds, i.e., with *m*/*z*. This shift increases with Δ *m*/*z* and thus with DP, but is already visible for DP2 with Δ *m/z* 18 for the two isotopologs. Further measurements of analytes in a wider *m*/*z* range beside this project revealed that TD at the maximum of a particular ion (TD_max_) linearly depends on *m*/*z* and also on Oct 2 DC. Oct 2 DC controls the kinetic energy with which the ions enter the trap. The higher the Oct 2 DC, the larger the RF voltage required to stabilize them. The maximum intensities achieved for each particular isotopolog do not show significant differences for DP3 and 4. For DP2, Me-*d*_*3*_-COS shows a larger maximum intensity than Me-COS, and the shape of the curve looks slightly different from the others due to the applied *Cap Exit* voltage, which is less appropriate for this low *m*/*z* range (449–467), as will be discussed below. For DP6, the situation is more complex due to the formation of double-charged ions. According to their smaller *m*/*z* values, these reach their maximum intensity at much lower TD values compared to the single-charged ions. Nevertheless, the trend mentioned above is also visible here. The maximum intensity of the single-charged COS is larger for the methylated compound, while its isotopolog shows a slightly higher intensity for the double-charged ions with the higher *m*/*z*.

The right column of Fig. 3 shows the normalized IR (Me-*d*_*3*_/Me). As can be expected from the slightly different intensity profiles discussed above, these values meet 1.0 at the intersection of the curves of the two isotopologs with IR < 1.0 before the intersection and > 1.0 beyond it. Since the difference between the isotopologs is smaller on the downward-sloping side, IR is nearly constant behind the crossing point, scattering around a value close to 1.1 under the conditions chosen for these measurements (e.g., Oct 2 DC 2.7 V). Different maximum intensities as observed for DP2 are assumed to be caused by discrimination effects during ionization or earlier stages of ion transport.

As long as the same maximum intensity is gained for both isotopologs (under the present conditions, this fits to DP3 and DP4), the highest accuracy would be attained by measuring the sample at the respective individual TD value under otherwise identical conditions. From a practical point of view, the values obtained at an optimized TD value close to the two maxima should be sufficient to obtain correct results.

In conclusion, the RF voltage at the ring electrode of the ion trap, set by TD, controls *m*/*z*-dependent ion storage in the trap with a selectivity that can already be observed at mass differences of the two border cases of Me-*d*_*3*_/Me isotopologs of COS. Due to the *m*/*z*-selective shift of the ion intensity maxima to larger TD values, IR/MR is < 1.0 at low TD, reaching 1.0 at the intersection of the shifted and distorted curves, while beyond this, they are slightly increasing. The effect increases with Δ*m*/*z*, i.e., with DP. As already mentioned, TD_max_ is shifted to lower values when the ions enter the trap with a lower kinetic energy due to applying a lower Oct 2 DC voltage. Therefore, for further experiments, TD values were chosen in a range where IR/MR looks robust and where effective trapping is expected even when the octopole DC voltage is changed (∆Oct 2 DC ~ ± 1.0 V). Thus, TD 66.1, 70.6, 78.0, 92.9, and 100.3 for DP2–6, respectively, were used in the subsequent steps of this investigation. It also turned out that applying the instrumental parameters common in MC analysis (s. above) causes IR/MR > 1.1 for DP2, decreasing with DP to IR/MR < 1.00 for DP6. This is not only the result of the deviation from the particular TD_max_, but also due to a difference of the individual maximum intensities achieved. These deviations from IR/MR 1.00 result from preceding steps of ion transportation or different ionization efficiencies. Therefore, in the following, we will look how the octopole voltage settings affect the transmission efficiency of ions depending on their *m*/*z*.

### Influence of octopole voltages on ion transportation

The most important adjustments for ion transport are the RF and DC voltages of the octopoles. Like the quadrupole, an octopole is a mass filter. However, in contrast to quadrupoles, multipoles show a better ion transmission with wide band pass characteristics at less defined *m*/*z* cutoff and more diffuse limits of ion stability [27]. When used for mass analysis, AC and DC voltages of quadrupoles are enhanced linearly to achieve a successive selective transmission for ions with increasing *m*/*z*. In a typical MC pattern analysis, ions in a wide *m*/*z* range shall pass non-selectively and reach the ion trap. The largest transmission efficiency is usually obtained when DC = 0. However, in order to enhance sensitivity and speed, the basis Oct 2 DC voltage is generally set to a minimum of 1.7 V in our instrument. Quadrupoles (and octopoles as well) are closely related to ion traps. Ions can only pass if they move on stable ion trajectories. For more detailed information on how the applied DC and RF voltage effect stable ion trajectories, the reader is referred to the literature [10].

In order to study discrimination effects during the ion transportation through the octopoles, we varied the Oct 2 DC and Oct RF based on the values suggested for a given TM in the *smart mode*. Beside the TD, the voltages of Oct 2 DC and Oct RF are controlled by the parameter *Target Mass* (TM) in the *smart mode*. As mentioned, Oct 2 DC is set to 1.7 V at TM 200 up to a TM of 500; this basic level of Oct 2 DC is kept constant, while Oct RF increases from 131 to 200 Vpp. When further raising TM up to 1500, RF is maintained at 200 Vpp, while DC is increased linearly up to 3.67 V (s. ESM Fig. S2). This means that with increasing TM, first the transmission for smaller *m*/*z* is progressively reduced by shifting the trajectory of stability to larger *m*/*z*, whereas from TM 600 on the transmission range becomes narrower, i.e., selectivity is enhanced in favor of larger *m*/*z*. The TD values for these studies were adjusted to the values given above and in Table 2. *Cap Exit* (280 V) and all other parameters were maintained as before.

**Table 2:** Final measurement parameters for the quantitative analysis of binary Me-/Me-*d*_*3*_-COS mixtures of DP2–6. Skimmer voltage 40 V; Oct RF 200 Vpp

DP	*Trap Drive*	Oct 2 DC [V]	*Cap Exit *[V]	IR (Me*-d*_3_/Me)/MR
2	66.1	2.48	110^a^	1.00 ± 0.03
3	70.6	2.70	280	1.00 ± 0.05
4	78.0	2.48	280	0.95 ± 0.03
5	92.9	2.24	280	1.04 ± 0.02
6	100.3	1.74	280	0.98 ± 0.04

^a^After the measurements presented in this paper, the instrument was serviced. When measurements were repeated, a shift of the TD maxima of individual *m*/*z* of our standard solutions was observed, before the measurement series (TD; Oct 2 DC Cap Exit) were reproducible over month. Little changes of the performance of a mass spectrometer are known depending on several parameters (voltage stability, space effects, quality of vacuum). However, the deviations of expert parameters after the service were beyond the day-to-day deviations and needed to be considered. When the optimal parameters were adjusted, results could be reproduced

^b^90 up to 210 V is appropriate

**Fig. 4:**
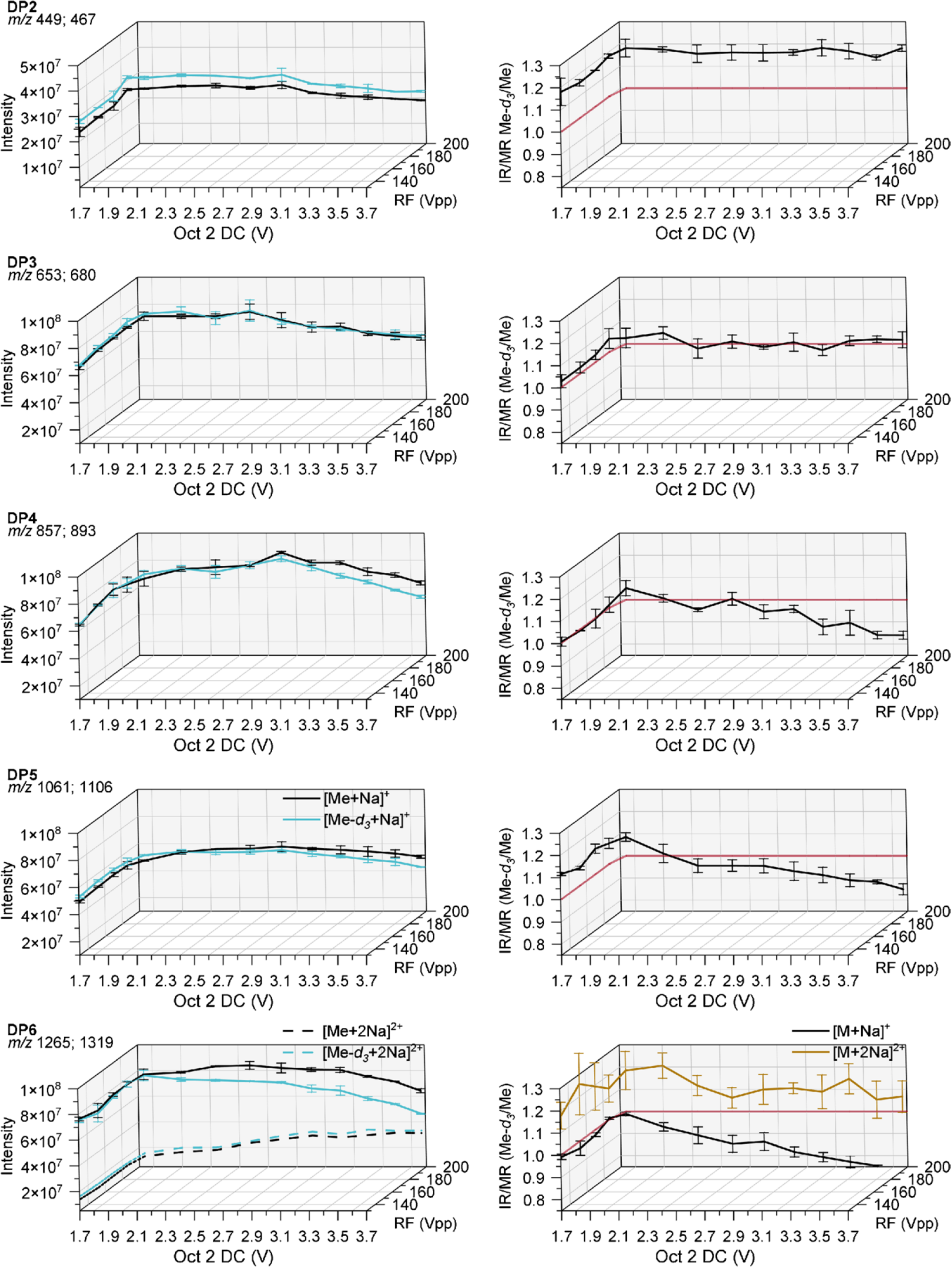
Left: absolute intensities recorded for the binary mixtures of Me-COS and Me-*d*_*3*_-COS at a total concentration of 2 · 10^−6^ M in MeOH by ESI-IT-MS (syringe infusion) at various Oct 2 DC and Oct 2 RF voltages. Right: calculated intensity ratio (IR). Data are corrected for the exact molar ratio (MR) according to the reference data given in Table 1 to represent an equimolar mixture. Further measurement parameters are given in Table 2; *n* = 3

Figure 4 shows the results in a 3D plot. On the *yz*-plane on the left, the ion intensities are displayed with an increase of RF voltage from 131 to 200 Vpp at a constant Oct 2 DC voltage of 1.7 V. On the *xy*-plane on the right, the subsequent increase of DC at constant RF of 200 Vpp is illustrated. In the graphic for DP6, single- and double-charged ions are included separately. The rise of Oct RF at constantly low Oct 2 DC (1.7 V) did not show a pronounced impact on ion intensities. Constant differences in favor of the deuterated isotopolog are still observed for DP2, resulting in IR/MR of 1.16 ± 0.03. This bias had already been observed independently on TD and obviously is neither caused by the octopole settings. For DP3–6, the IR is slightly above 1.0, now measured at the selected TD. While keeping the Oct RF plateau at 200 Vpp, Oct 2 DC was further increased according to larger TM. As visible in the *xy*-plane of Fig. 4, the absolute ion intensities decrease above a certain DC voltage after first keeping a constant level (DP2 and 3), or after a slight but steady increase (DP4–6). For these higher masses (DP4: *m*/*z* 857 and 893; DP5: *m*/*z* 1061 and 1106; DP6: *m*/*z* 1265 and 1319), the intensity of Me-*d*_*3*_-COS decreases faster than that of its Me-isotopolog with increasing Oct 2 DC with an intersection (IR = 1.0) at around 2.0 V, beyond which the lighter isotopolog (Me-COS) more and more dominates. Since the kinetic energy of the ions in axial direction increases with Oct 2 DC and causes a TD shift to higher TD_max_ values as mentioned above, the chosen TD value is no longer appropriate to stabilize the heavier isotopologs as well in the ion trap. The visible drop in intensity is more attributed to the TD shift than to the discrimination effects during the ion transport.

For DP6, again, double-charged ions must be considered in addition to single-charged ones. Both types behave similarly, when only the RF voltage is raised. However, when subsequently ramping Oct 2 DC, the intensity of the double-charged ones increases while that of the single-charged ions decrease. When the measurements for DP6 were recorded at the TD_max_ for the doubly charged ions, the same curve shape is obtained as for DP3 (s. ESM Fig. S5). This observation corresponds with the fact that the double-charged ions of DP6 have approximately the same *a* and *q* parameters as DP3 and therefore perform the same ion motion in the octopole (DP3^+^: *m*/*z* 653 and 680; DP6^2+^: *m*/*z* 644 and 671). As seen above, in the case of [M+2Na]^2+^, Me-*d*_*3*_-COS dominates, while for [M+Na]^+^, the lighter Me-COS are favored. This is because of the selected TD value (see Fig. 3).

In conclusion, the octopole causes bias as a mass filter at too-large DC voltages for COS with *m*/*z* > 800 (≥ DP4), while RF did not show a significant impact on IR/MR in the studied range. This partly results from the shift of the optimal TD (RF of the ion trap) for maximum intensity, with increasing Oct 2 DC. DP2 and 3 are not affected; however, DP2 still shows a constant deviation from the real IR. As before, robust areas or areas where the IR/MR was close to 1.00 were chosen to find the optimal voltages (Table 2). Where feasible, preference was given to higher RF and DC voltages, i.e., enhanced selectivity and signal intensity. High signal intensities for the higher DPs are particularly advantageous for MC samples. Furthermore, there is a risk that the trap will be overloaded at low RF and DC voltages in case of a MC (highest transmission).

### Influence of Cap Exit and Skimmer voltage on ion transportation

From the ESI source at atmospheric pressure, the ions are transferred to the first low-pressure area via a transfer capillary. Between the exit of this capillary and the first octopole, a skimmer is located (Fig. 2). Page et al. [28] studied the process of the ion transmission through the capillary in more detail. High temperatures promote ionization efficiency by improved desolvation, but simultaneously, the mobility of the ions increases and consequently the probability to strike the wall. A destruction of clusters via ion transfer is possible through collision-induced dissociation (CID) by the applied potential difference between the capillary outlet (nozzle) and the skimmer—the so-called nozzle skimmer CID. Lower potential differences effect dissociation of non-covalent complexes, while—depending on the analytes’ stability—higher ones can cause fragmentation of covalent bonds in this relatively high-pressure area [15, 19]. At the same time, the applied voltage between the capillary outlet (*Cap Exit*) and the skimmer is the driving force for the ion transfer in the high-vacuum area of the instrument.

All measurements presented so far have been performed at a *Cap Exit* of 280 V. In the *smart mode*, *Cap Exit* is set by the parameter *Compound Stability* (CS) between 0 and 1000%. The *Cap Exit* voltage increases with CS while the slope of this increase depends on the selected TM (s. ESM Fig. S3). The higher the TM, the steeper the slope. At TM 1000, used for MC analysis so far, *Cap Exit* reaches its final value of 280 V already at 400% CS. In contrast, at TM 400, the ultimate *Cap Exit* value is reached at 800% CS. For the following experiments, *Cap Exit* was set directly in the *expert mode*. Based on the preliminary results, TD and Oct 2 DC/Oct RF were adjusted as listed in Table 2.

**Fig. 5:**
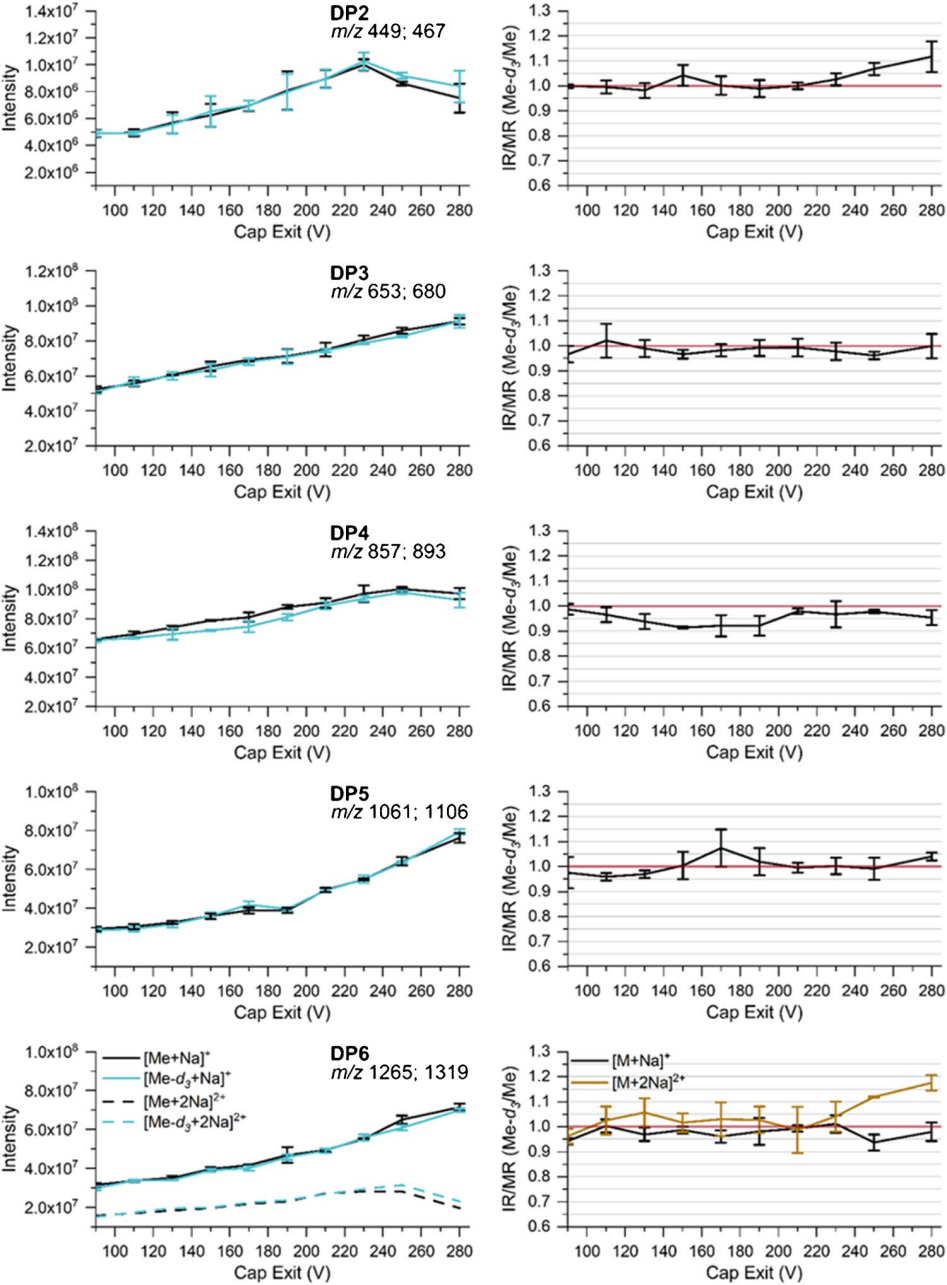
Left: absolute intensities recorded for the binary mixtures of COS (Me and Me-*d*_*3*_) at a total concentration of 2 · 10^−6^ M in MeOH by ESI-IT-MS (syringe infusion) at various *Cap Exit* voltages. Right: calculated intensity ratio (IR). Data are corrected for the exact molar ratio (MR) according to the reference data given in Table 1 to represent an equimolar mixture. Further measurement parameters are given in Table 2; *n* = 3

Figure 5 shows how the ion intensities develop with the *Cap Exit* voltage, while the skimmer is kept at 40 V. On the one hand, the increase visible for all DP is due to the increasing potential difference between the *Cap Exit* and skimmer (improved ion transportation), and on the other hand due to CID of double-charged adducts (DP4–6) or dimeric clusters (DP2).

Except for DP2, no significant difference for the isotopologs is observed. As seen above (Fig. 3 and Fig. 4), IR/MR for DP2 was always around 1.15 for all octopole settings and the robust TD range. We now recognize that this discrimination of Me-COS is due to the high *Cap Exit* of 280 V selected for the former experiments. At *Cap Exit* up to 210 V, no discrimination is visible. Ion intensities are equal and IR/MR is close to 1.0. However, in this range, all types of uniform and mixed clusters of the type [2M+Na]^+^ are detected for DP2 which were not considered in the evaluation. This should not impair the results since complexation behavior of the isotopologs was equal on average. Nevertheless, for application to MCs with their larger complexity, any formation of additional ion species is unwanted. The amount of dimeric clusters decreases with increasing *Cap Exit* voltage due to CID from 20% (90 V) to 0% (230 V) referred to the target ion [M+Na]^+^. At 230 V and beyond, clusters were no longer detected. However, at this point, the ion intensities of the isotopologs start to decrease and diverge with the heavier isotopolog becoming superior. A similar course, albeit less pronounced, is observed for double-charged DP6. With increasing *Cap Exit* voltage, field strength increases and thus the kinetic energy of the ions. Ions of lower *m*/*z* gain larger velocities than those of higher *m*/*z*, and therefore, their probability to strike the wall is more enhanced. The absolute difference of velocities is the larger the smaller *m*/*z* (due to $v\sim \sqrt{z/m}$). Furthermore, in case of collisions, the larger molecules (DP > 2) have more possibilities of energy dissipation and thus of “surviving.” In contrast, DP2 is the only COS with only one glycosidic linkage and thus is conformationally less flexible. This will also influence sodium complex stability. Double-charged adducts were also detected for DP4 (10% ➔ 0% at 230 V) and DP5 (15% ➔ 10% at 280 V); however, with respect to their comparable relative amounts, they were not considered in the evaluation.

In conclusion, *Cap Exit* (or CS) should not be adjusted too high for DP2 (90–210 V). For all other DPs, we chose 280 V, in order to suppress double-charged adducts as far as possible, which might interfere with the evaluation of more complex MC samples. And high *Cap Exit* will promote high intensities of the higher DPs, which are normally present in small amounts in a real MC sample.

Finally, Table 2 summarizes the measurement conditions specified for each DP. In addition, the IR/MR values obtained under these conditions are listed.

### Concentration-dependent ESI-MS measurements

So far, we have focused on possible bias during ion transportation and storage, presuming that the two isotopologs are chemically thus similar that they yield equal ionization in the ESI process. However, as outlined in the introduction, CH_3_ and CD_3_ are quite more different than other isotopolog groups with a more compact structure of the deuterated forms. To find out whether there are discrimination effects because of different ionization efficiencies, we measured a serial dilution of each binary mixture.

The ESI process has been studied and discussed in detail by various groups [13–16, 29–34]. Briefly: By spraying the sample solution in a high electric field, charged droplets are formed with the excess of charge on its surface. Shrinking of droplets due to loss of solvent repeatedly provokes Coulomb explosions with a much higher chance for the ions located in the droplet surface layer to “survive” in the next droplet generation. As mentioned in the “Introduction,” the partition equilibria of analytes and background electrolytes of various chemistry and concentration are important for ion-suppression effects [13, 14, 16, 29]. When the surface becomes saturated, in normal ESI at about *c* = 10^−5^ M, competition of various ions for the surfaces becomes relevant, whereas the less polar compounds with the larger surface activity predominate [35]. This can cause complete suppression of other analytes, even if present at comparable concentrations. Consequently, below saturation, two charged analytes should show equal sensitivity and linear increase of the ion current with concentration as long as they are not too different regarding their chemistry [13]. In the saturated regime, the ion with the higher sensitivity (for instance, due to surface activity) will predominate and a continuous re-supply from the bulk of the droplet after the depletion of ions from the surface will take place.

The sodium complexation ability (or relative stabilities of the [COS+*n* · Na]^*n*+^) should also be considered [11, 12]. According to Sherman and Brodbelt [14], the involved equilibria for Na^+^ complexation of an analyte A in combination with the equilibria of all species between the bulk and droplet surface can be described as follows:

6 $${K}_{{bs}}=\frac{{\left[A-Na^{+}\right]}_s}{{\left[{A}\right]}_s{\cdot}{\left[{{Na}}^{+}\right]}_s}=\frac{{K}_A{\cdot}{K}_{Na}}{{K}_{A- Na}}{\cdot}{K}_{{bi}}$$

with indices b for binding, s for surface, and i for inner part of the droplet. *K*_bi_, *K*_bs_, *K*_A-Na_, etc. describe the distribution of A-Na^+^ between the surface and inner droplet, respectively, e.g.

7 $${K}_{{A}-{Na}}=\frac{{\left[A-Na^{+}\right]}_s}{{\left[A-Na^{+}\right]}_{{i}}}$$

The system of equations, additionally considering a background electrolyte, was numerically solved for various assumptions [14]. The ion intensity of the charged complex in dependence on the analyte concentration showed the typical linear increase and flattening beyond saturation. With an increasing amount of background electrolyte, the intensity of the analyte decreased due to a competitive effect; hence, the saturation point is shifted to a higher analyte concentration and the linear range extends over a wider concentration range. Variation of the complexation constant *K*_b_ also influences the course of the concentration-dependent ion intensity: the higher the *K*_b_, the larger the intensity at low concentration of the analyte, but finally converging at the maximum intensity (due to saturation) [14].

A concentration-dependent measurement of the equimolar binary COS mixtures should indicate whether any bias occurs below (due to *K*_b_) and above the saturation point (due to *K*_s_). All Me-*d*_*3*_/Me-COS mixtures were measured in the range of approximately 10^−9^ M to 2 · 10^−4^ or 2 · 10^−3^ M (DP2) (total concentration of the COS-Me and Me-*d*_*3*_) in MeOH at the selected settings for TD, octopole, and *Cap Exit* as summarized in Table 2.

**Fig. 6:**
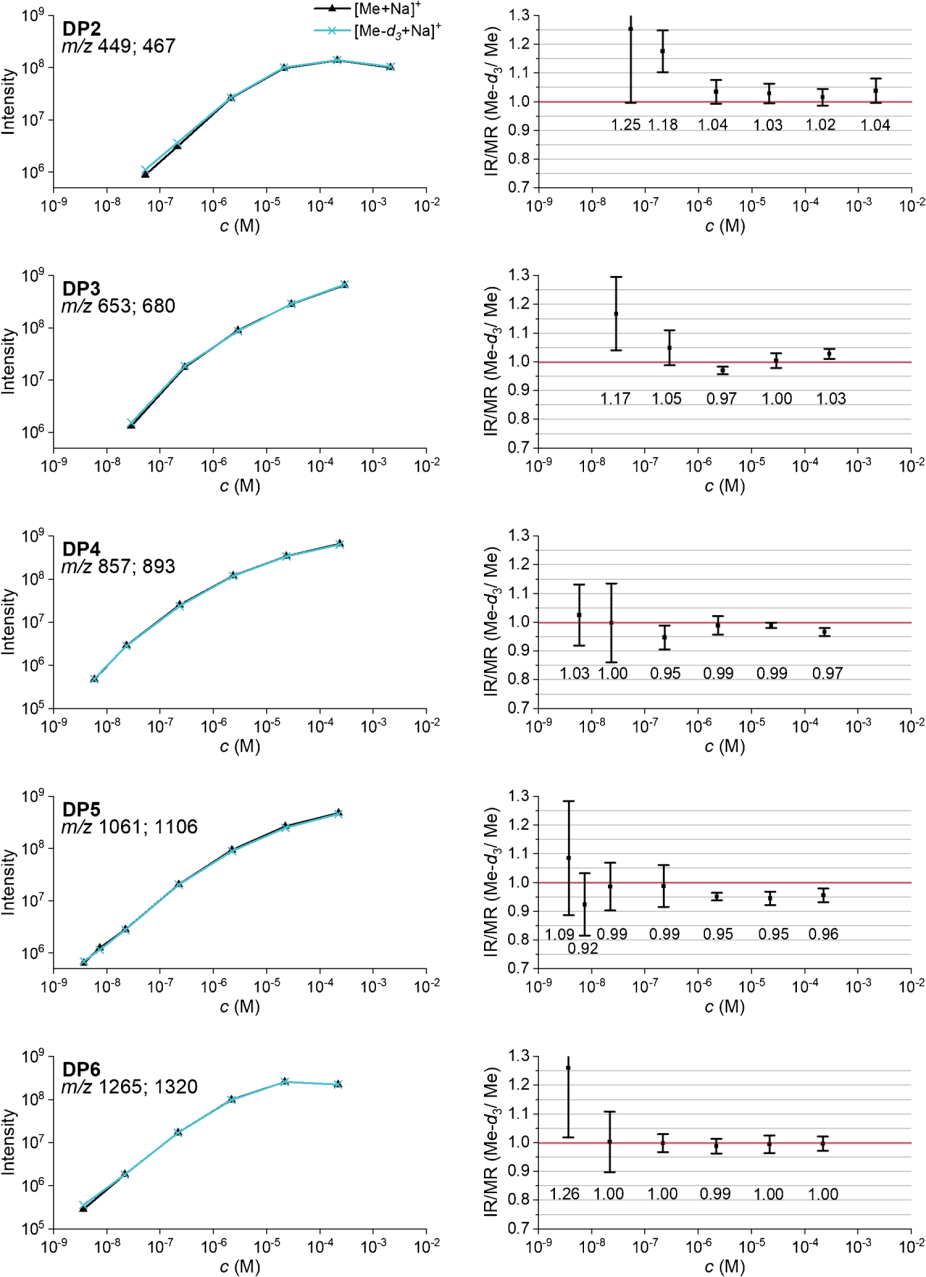
Left: absolute intensities of the serial dilution of the binary mixtures of COS (Me and Me-*d*_*3*_) in MeOH by ESI-IT-MS (syringe infusion). Right: calculated intensity ratio (IR). Data are corrected for the exact molar ratio (MR) according to the reference data given in Table 1 to represent an equimolar mixture. Further measurement parameters are given in Table 2; *n* = 5

Figure 6 shows the results. The double-logarithmic plots for both isotopologs show the expected linear increase and finally flattening due to saturation. They are congruent for all DP. The IR values scatter around 1.00 for most of the COS measurements, even above the saturation regime. As a result, it can be concluded that both analytes have the same chemical properties relevant for *K*_bs_ (according to (6)) and there are no displacement effects with increasing concentration. As expected, the standard deviation is larger for the IR obtained from the very diluted samples. Thus, the slight physicochemical differences known for CH_3_ and CD_3_ groups mentioned above obviously do not cause different ion yields in the ESI process.

To check the reproducibility, the measurements were repeated in the range of 10^−7^–10^−5^ M on two other days with 5 measurements each. The *intraday* standard deviation increased with decreasing concentration and was between 0.01 (1%) and 0.07 (7%), with one exception. The *interday* standard deviations for this concentration range were between 0.00 (0%) and 0.05 (5%) (for data s. ESM, Table S1).

Finally, the normalized intensity ratio IR/MR was determined from all measurements that were measured during the optimization steps under the later-specified optimal measurement parameters and for a concentration between 10^−7^ and 10^−5^ M (Table 3). It was calculated as weighted average, and the internal as well as external SD was determined according to formula (1)–(3). The determination of the uncertainty budget was carried out according to type A evaluation. The SD of the HPLC reference analysis as well as of the MS measurements were taken into account (always the larger deviation of internal and external SD). A *t*-distribution was assumed and a confidence interval of 95 %.

**Table 3:** Intensity ratio IR (Me-*d*_*3*_/Me)/MR) given with expanded uncertainty *U* (95 % confidence)

	**DP2**	**DP3**	**DP4**	**DP5**	**DP6**
**IR/MR**	1.040 ± 0.009	0.995 ± 0.004	0.979 ± 0.004	0.971 ± 0.008	0.993 ± 0.006

Ratios are a weighted average of 10–12 values per DP, which were received from triplicate and quintuple measurements.

The IR/MR ratio is close to 1.0 for all DPs. In a previous study, we have investigated the ratio of ions of binary mixtures of stereoisomeric *O-*Me and *O*-Me-*d*_*3*_-maltooligosaccharides (MOS) [36] and found an intensity ratio IR/MR slightly above 1.0 for DP2 (1.01 ± 0.04) which non-linearly decreased with the DP to 0.78 ± 0.02 for DP6. These measurements were carried out with instrumental parameters usually applied by us for real MC samples (TM 1000, TDL 100%, and CS 1000%; *Cap Exit* 280 V; Oct 2 DC 2.7; Oct RF 200 Vpp). As we have recognized now for the corresponding COS derivatives, this trend it is not caused by discrimination effects during the ionization, but rather during the transportation as well as storage of ions in the trap.

### Application to methyl cellulose

After having studied all potential sources of bias in ESI-IT-MS for the two border cases of a Me/Me-*d*_*3*_ profile, i.e., the uniformly *O*-methylated and *O*-deuteromethylated COS, respectively, the selected conditions were applied to real samples, two MCs with DS 1.29 (MC 1) and 1.96 (MC 2). The MCs were perdeuteromethylated, partially hydrolyzed, and measured by syringe pump infusion (*c*_total_ 5 · 10^−5^ M) in MeOH under the conditions found most appropriate for each particular DP (expert conditions, Table 2). For comparison, the samples were also measured under the standard conditions usually applied in the *smart mode* at TM 1000 [36]. The relative intensities of all constituents belonging to one DP, each with one Me less and one Me-*d*_*3*_ more than the preceding one (e.g., in the case of DP2 from Me_6_/(Me-*d*_*3*_)_0_ over Me_5_/(Me-*d*_*3*_)_1_, Me_4_/(Me-*d*_*3*_)_2_ etc. up to Me_0_/(Me-*d*_*3*_)_6_) and Δ*m*/*z* 3, are corrected for their isotopic compositions and then normalized to 100%.

**Fig. 7:**
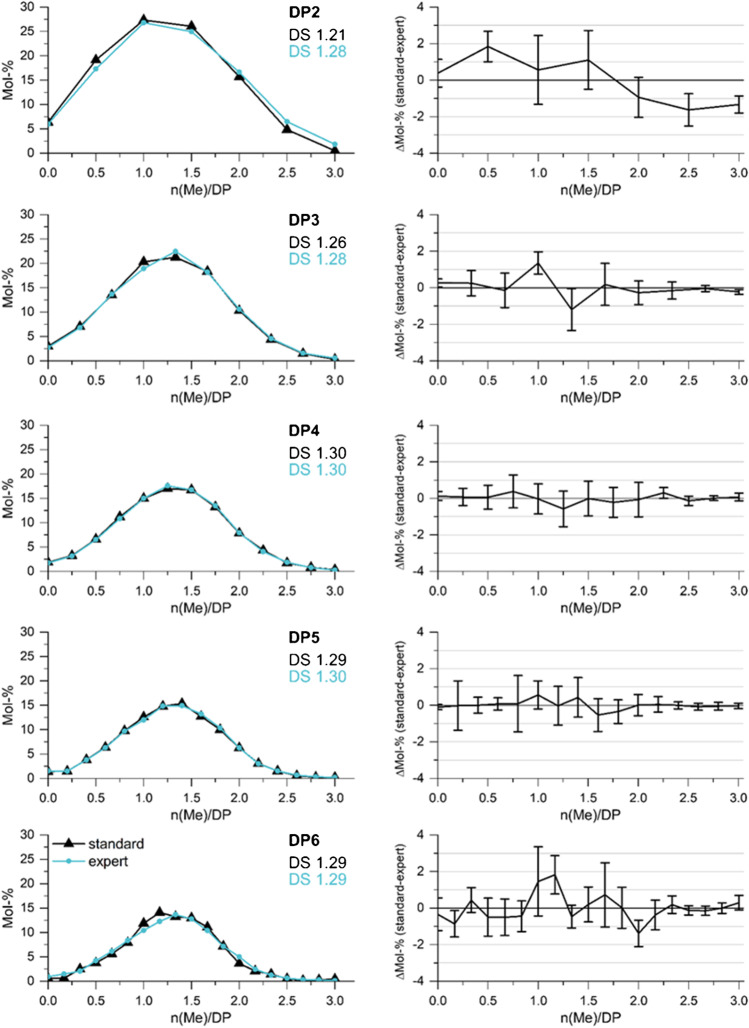
Left: methyl distribution in COS obtained by partial hydrolysis of perdeuteromethylated MC1 (DS 1.29) by ESI-IT-MS measured by syringe pump infusion under standard conditions (*Cap Exit* 280 V; Oct 2 DC 2.7 V; Oct RF 200 Vpp) and expert conditions (see Table 2). Right: differences between the results obtained under these two conditions; *n* = 5

Results for MC 1 are shown in Fig. 7 (for MC 2 s. ESM Fig. S6). The left column presents the results for the methyl substituent distribution analyzed under standard and expert conditions (Table 2). The profiles look very similar, independent of the instrumental parameters applied. That the differences are within standard deviations can be seen in more detail in the right column. Significant differences are only observed for DP2. The main parameter of control of plausibility of results is the average DS/DP calculated from the MS data. It should be constant for each DP and in agreement with the average DS of the entire material. DS calculated from measurements under conditions optimized for each particular DP are all close to the average DS of 1.29. Those obtained under standard conditions show a slight trend of increasing DS with DP, which is in agreement with the discrimination of the deuteromethyl-rich COS when TD is not optimized, but the effect is very small. For MC2, the same DS trend could be observed (s. ESM Fig. S6).

These results confirm that quantitative analysis of real MC samples by ESI-IT-MS of derived *O*-Me/*O-*Me-*d*_*3*_ isotopologs, for which the most abundant analytes are more similar to each other regarding chemistry and their *m*/*z*, does not significantly suffer from the application of conditions which are not optimized for each particular DP, but nevertheless appropriate. The impact of these common settings, for instance the high *Cap Exit* value and the large Oct 2 DC, is best visible for DP2 of MC1, for which the DS (1.21) is significantly too low, according to the overestimation of the preferentially deuteromethylated cellobioses. With increasing DP, the methyl distributions become narrower, and thus, the analytes significantly contributing to the profile become more similar. Thus, slight discrimination effects observed for the border cases can be neglected in the case of conventional MCs. However, they have to be considered in the case of bimodal [37] or block-like structures [5], i.e., in the case of main contributions of less similar constituents or even the extreme cases of a DP profile.

## Conclusion

Our study of the impact of instrumental parameters in ESI-IT-MS analysis of *O*-Me and *O*-Me-*d*_*3*_-COS showed various sources of discrimination, mainly related to the *m*/*z* differences of the isotopologous analytes. While ionization as sodium adducts did not show any bias in spite of slight chemical differences of these special types of isotopologs, ion transportation and ion storage turned out to be more critical. No concentration dependence of the intensity ratio (IR/MR) was found in the range 10^−9^ to 10^−3^ M. The voltage at the transfer capillary exit (*Cap Exit*) becomes critical only for ions of low *m*/*z* (< 500, DP2), probably due to too strong acceleration of the lighter ions, enhancing the probability of selective losses by striking the wall and larger sensitivity to dissociation than their higher homologs. Parameters controlling ion transportation, mainly octopole DC and RF voltages, had to be adjusted according to a compromise between sensitivity and selectivity. The lower the octopole DC voltage, the less selective ion transport is. Finally, the *Trap Drive* value (TD, controlling RF voltage) at which maximum ion intensities are obtained (TD_max_) are shifted to larger RF voltages with increasing Oct 2 DC and increasing *m*/*z*, i.e., to higher values for the heavier Me-*d*_*3*_-isotopologs. By choosing an appropriate TD, these impacts can be leveled, and the former observed decrease of IR (Me-*d*_*3*_/Me) for MOS with DP can be avoided. Discrimination effects by the amplification process in the detector can presumably be neglected. Normalized IR/MR (i.e., corrected for the independently determined molar ratio MR of the analytes) between 0.971 ± 0.008 and 1.040 ± 0.009 were obtained with no DP-related trend. Application of the instrumental parameters selected for each particular DP to common MCs showed that the usually applied standard conditions are sufficient for these type of samples since their substitution profiles consist of a narrower distribution of more similar (*O*-Me)_m_-(*O*-Me-*d*_*3*_)_n_ COS. However, in the case of bimodal and block-like methylated samples, the border cases play a bigger role. Consequently, for those, the better-adjusted instrumental parameters should be applied. For chemically more different cellulose ethers as for instance hydroxyethyl methyl celluloses (HEMC), the findings from this study will be conducive for improving quantitative MS analysis. Respective studies are under progress.

## Supplementary Information

ESM 1 (PDF 817 kb)
