# Supplementary material for: Impact of instrumental settings in electrospray ionization ion trap mass spectrometry on the analysis of multi-CH3-/CD3-isotopologs in cellulose ether analysis: a quantitative evaluation
Source: Anal Bioanal Chem. 2021 Dec 16;414(3):1279–96. doi: 10.1007/s00216-021-03767-w (PMC8724165; doi:10.1007/s00216-021-03767-w)
Supplement: Supplementary file 1 — (PDF 817 kb) [file 216_2021_3767_MOESM1_ESM.pdf]

Analytical and Bioanalytical Chemistry

Electronic Supplementary Material

Impact of instrumental settings in electrospray ionization ion trap mass spectrometry on the analysis of multi-CH_3-/CD_3-isotopologs in cellulose ether analysis: a quantitative evaluation

Sarah Schleicher, Inka-Rosalia Lottje, Petra Mischnick

Institute of Food Chemistry, Technische Universität Braunschweig, Schleinitzstr. 20, D-38106 Braunschweig, Germany

Corresponding author: Petra Mischnick, p.mischnick@tu-braunschweig.de

ORCID, Petra Mischnick: 0000-0002-8313-3313

A) Smart parameters

Measurements were performed with Bruker Ion Trap HCT Ultra ETD II. The instrument has two measurement modes. The *smart mode* with the parameters *Target Mass* (TM), *Compound Stability* (CS) and *Trap Drive Level* (TD-Level). These parameters adjust indirectly the voltages of the transfer capillary (*Cap Exit*), the RF voltages of the octopoles and the DC voltage of the second octopole (Oct 2 DC) as well as the amplitude of the ion trap (*Trap Drive*). In the *expert mode* it is possible to adjust these parameters directly. The following graphics show the correlation between *smart* and *expert mode* for the positive ion modus.

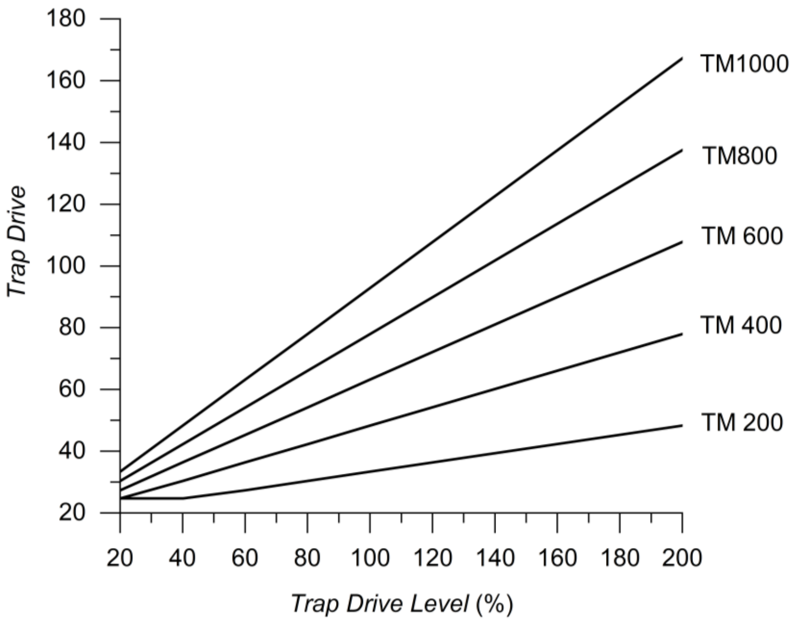

Fig. S1 *Trap Drive* (TD) related to the selected *Trap Drive Level* for different *Target Masses (*TM*)* for Bruker HCT Ultra ETD II.

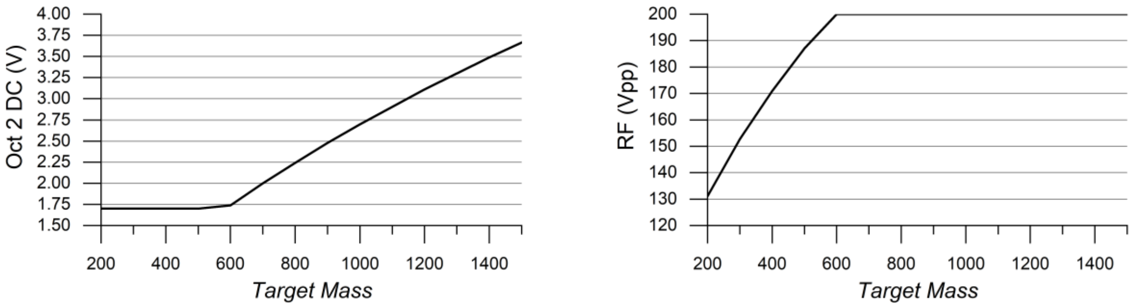

Fig. S2 Octopole voltages Oct 2 DC and Oct RF related to the selected *Target Mass* (TM) in the *smart mode* of the Bruker HCT Ultra ETD II

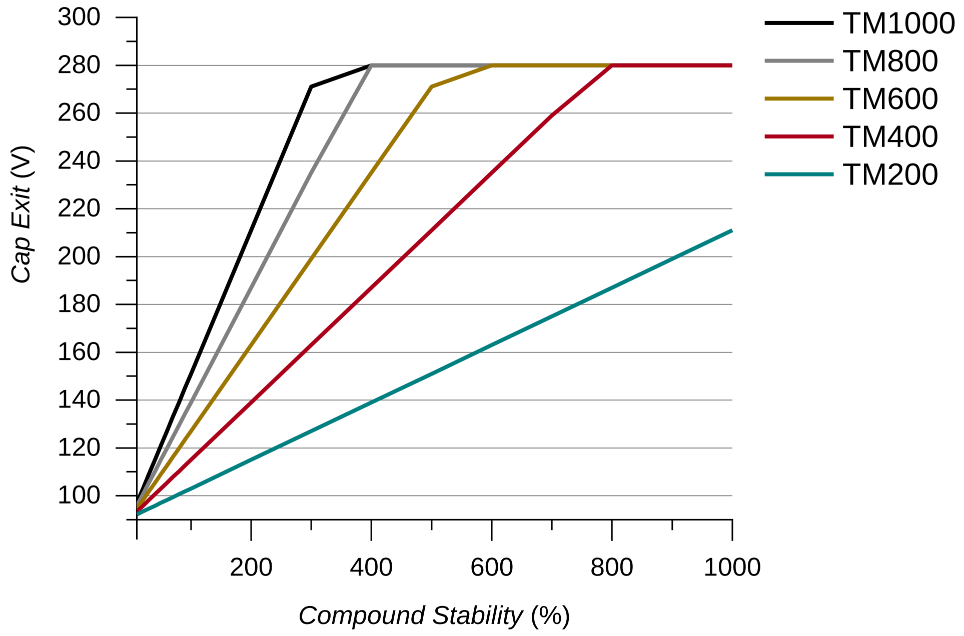

Fig. S3 Set of *Cap Exit* for a chosen *Target Mass* and *Compound Stability* in the *smart mode* of the Bruker Ion Trap HCT Ultra ETD II

B) Structure of the Analytes

In Fig. S4 the structures of the analytes of the binary mixtures are presented as well as their *m/z*-ratio as $[M+Na]^+$-adducts.

DP2
m/z 449 ; 467

DP3
m/z 653; 680

DP4
m/z 857; 893

DP5
m/z 1061; 1106

DP6
m/z 1265; 1319

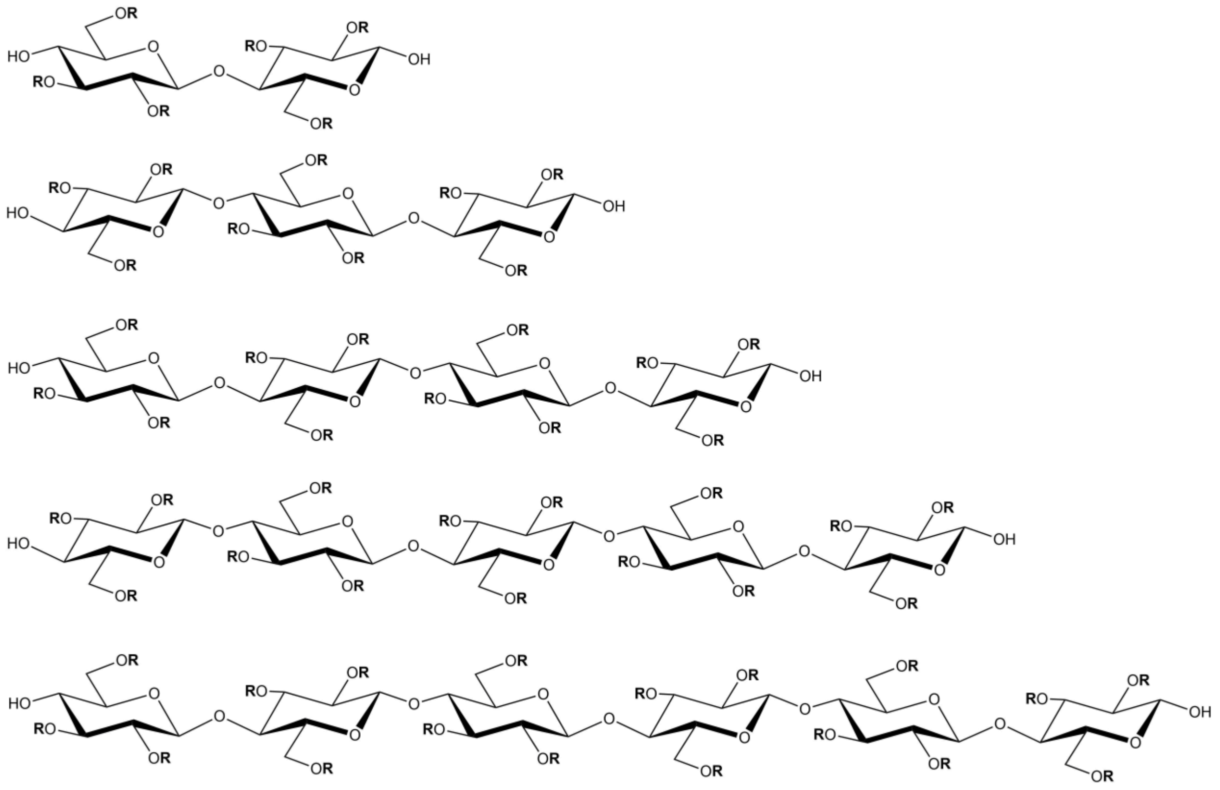

Fig. S4 Structures of the analytes of the binary mixtures of fully methylated (R= CH_3) and deuteromethylated (R= CD_3) cellooligosaccharides

C) Influence of the octopole voltages on double-charged ions

For COS of DP6, beside single-charged ions, also double-charged ions $[M+2Na]^{2+}$ were detected. Fig. S5 shows their intensities measured in dependence on the octopole voltages at the TD value (70.6) selected for DP3. The *m/z* of the double-charged ions of DP6 (644; 671) are close to the single-charged ions $[M+Na]^+$ of DP3 (653; 680), compare **Fig. 4** body text. The ions have therefore more or less the same *a*- and *q*-parameters and should perform the same ion motion in the octopoles.

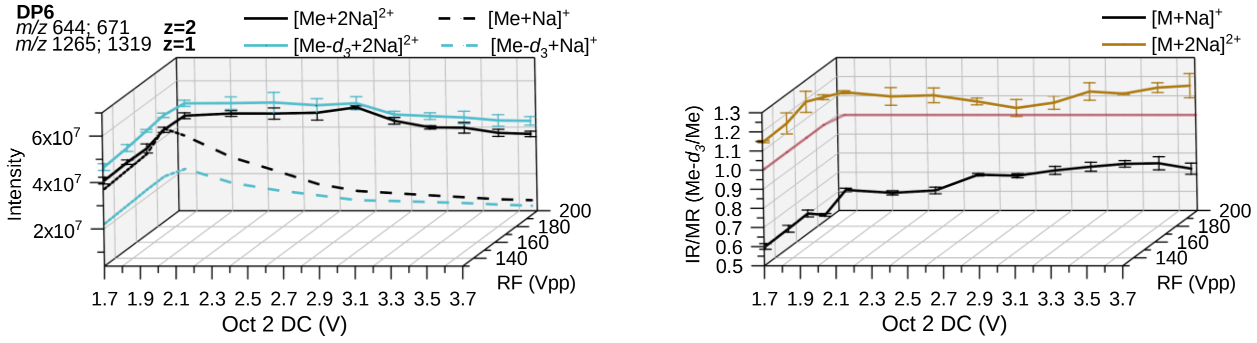

Fig. S5 Left: absolute intensities recorded for the binary mixtures of Me-COS and Me-d_3-COS at a total concentration of $2{\cdot}10^{-6}$ M in MeOH by ESI-IT-MS (syringe infusion) at various Oct 2 DC and Oct 2 RF voltages. Right: calculated intensity ratio (IR). Data are corrected for the exact molar ratio (MR) according to the reference data given in Table 1 (body text) to represent an equimolar mixture. Further measurement parameters are given in Table 2, body text. TD was 70.6 and n=3

D) Reproducibility of the IR/MR

To prove the reproducibility of the IR(Me-d_3/Me)/MR), the binary mixtures were measured at three different concentrations five times on three days. The *intraday*, as well as the *interday,* mean and standard deviation were determined (Tab. S1).

Table S1 Reproducibility of the IR(Me-d_3/Me)/MR) of the binary mixtures

DP	Concentration (M)	*Intraday* mean ± SD (n= 5)			*Interday* mean ± SD (n=5; p=3)
		Day 1	Day 2	Day 3	
2	10^{-5}	1.03 ± 0.03	1.05 ± 0.04	1.05 ± 0.02	1.04 ± 0.01
	10^{-6}	1.04 ± 0.04	1.06 ± 0.05	1.09 ± 0.05	1.06 ± 0.03
	10^{-7}	1.18 ± 0.07	1.23 ± 0.20	1.28 ± 0.20	1.23 ± 0.05
3	10^{-5}	1.00 ± 0.03	1.04 ± 0.04	1.03 ± 0.02	1.02 ± 0.02
	10^{-6}	0.97 ± 0.01	0.99 ± 0.01	0.99 ± 0.04	0.99 ± 0.01
	10^{-7}	1.05 ± 0.06	1.05 ± 0.06	1.05 ± 0.03	1.05 ± 0.00
4	10^{-5}	0.99 ± 0.01	0.96 ± 0.02	0.96 ± 0.02	0.97 ± 0.02
	10^{-6}	0.99 ± 0.03	0.97 ± 0.02	0.98 ± 0.03	0.98 ± 0.01
	10^{-7}	0.95 ± 0.04	0.96 ± 0.03	0.95 ± 0.06	0.95 ± 0.01
5	10^{-5}	0.95 ± 0.02	0.97 ± 0.04	0.93 ± 0.04	0.95 ± 0.02
	10^{-6}	0.95 ± 0.01	0.97 ± 0.02	0.94 ± 0.03	0.95 ± 0.01
	10^{-7}	0.99 ± 0.07	0.98 ± 0.06	0.96 ± 0.04	0.98 ± 0.01
6	10^{-5}	1.00 ± 0.03	1.02 ± 0.04	1.01 ± 0.03	1.01 ± 0.01
	10^{-6}	0.99 ± 0.03	1.00 ± 0.03	1.00 ± 0.01	0.99 ± 0.01
	10^{-7}	1.00 ± 0.03	0.99 ± 0.05	0.99 ± 0.03	0.99 ± 0.01

E) Substituent Distribution of Methylcellulose RAD29

The substituent distribution of methylcellulose RAD29 (MC2) was analyzed under the standard conditions (TM1000; *Compound Stability* 1000 %; *Trap Drive Level* 100 %) and the optimized-expert conditions (see Table 2 body text). The substituent distribution of both measurement parameters are shown in Fig 6 as well as their deviations from each others.

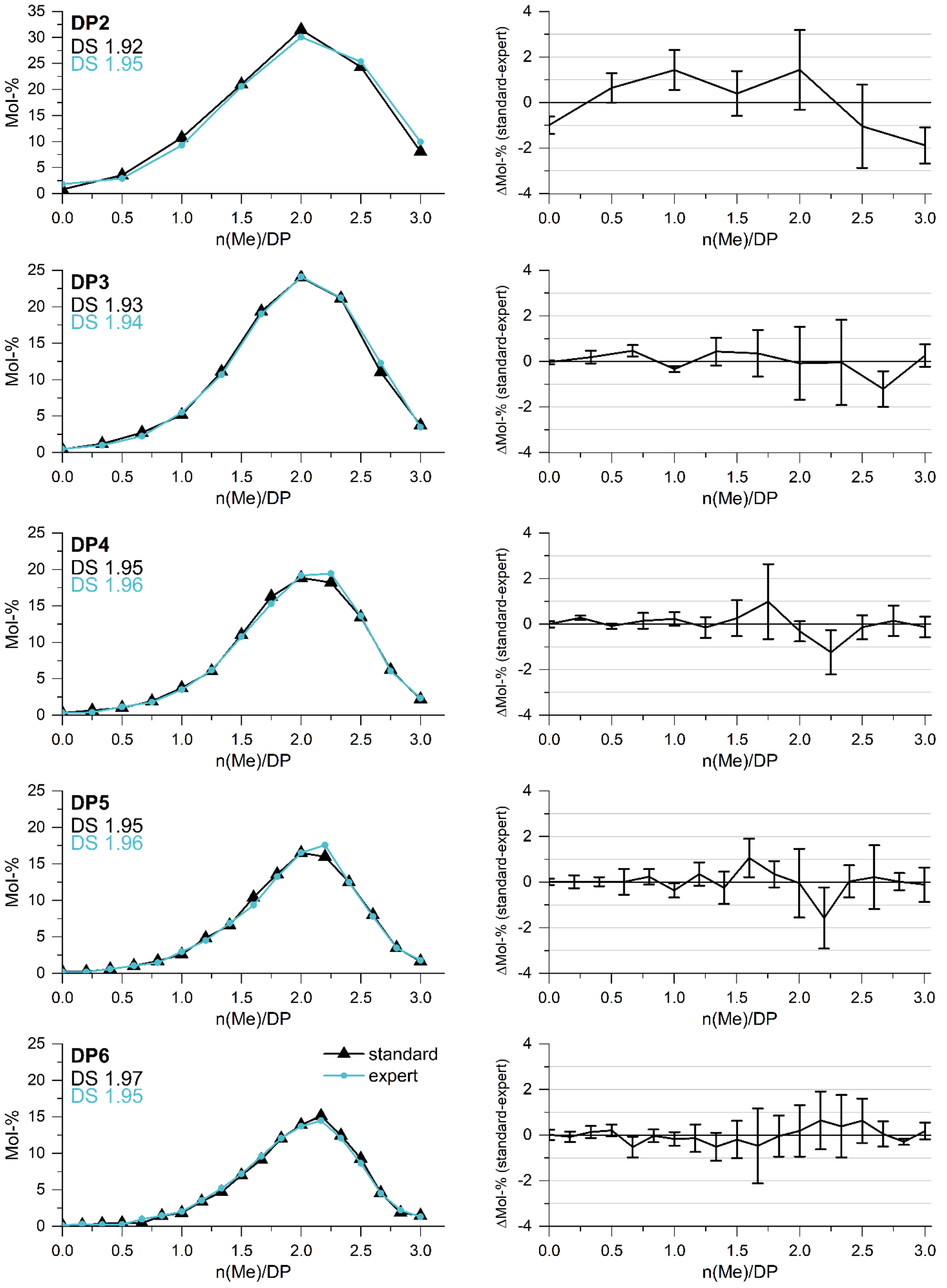

Fig. S6 Left: methyl distribution in COS obtained by partial hydrolysis of perdeuteromethylated MC2 (DS 1.96) by ESI-IT-MS measured by syringe pump infusion under standard conditions (*Cap Exit* 280 V; Oct 2 DC 2.7 V; Oct RF 200 Vpp), and expert conditions (see Table 2, body text). Right: differences between the relative intensities recorded under the two conditions; n= 5
